# Synthesis, structure and Hirshfeld surface analysis of 2-oxo-2*H*-chromen-6-yl 4-*tert*-butyl­benzoate: work carried out as part of the AFRAMED project

**DOI:** 10.1107/S2056989023011052

**Published:** 2024-01-05

**Authors:** Patrice Kenfack Tsobnang, Eric Ziki, Soso Siaka, Jules Yoda, Seham Kamal, Adam Bouraima, Ayi Djifa Hounsi, Emmanuel Wenger, El-Eulmi Bendeif, Claude Lecomte

**Affiliations:** a Chemistry Department, University of Dschang, PO Box 67, Dschang, Cameroon; b Physics Department, Faculty of Science of Structure of Matter and Technology, Université Félix Houphouet-Boigny, Abidjan, 08 BP 582 Abidjan, Cote d’Ivoire; c Chemistry Department, Faculty of Science, University of Nazi BONI, 01 BP 1091 Bobo Dioulasso 01, Burkina Faso; d Department of Medicine, Traditional Pharmacopeias and Pharmacy, Institute for Health Sciences Research, 03 BP 7192 Ouagadougou 03, Burkina Faso; e Physics Department, Faculty of Science, Cairo University, 12613 Giza, Egypt; f Chemistry Department, Faculty of Science, Masuku University of Science and Technology, Franceville, Gabon; g Physics Department, Faculty of Science, University of Lomé, Togo; h CRM2, CNRS Université de Lorraine, Vandoeuvre-lès-Nancy CEDEX BP 70239, France; University of Aberdeen, United Kingdom

**Keywords:** coumarin derivative, Hirshfeld surface, herringbone packing, crystal structure

## Abstract

In the title compound, C_20_H_18_O_4_, the dihedral angle between the 2*H*-chromen-2-one ring system and the phenyl ring is 89.12 (5)°. In the crystal, the mol­ecules are connected through C—H⋯O hydrogen bonds to generate [010] double chains that are reinforced by weak aromatic π–π stacking inter­actions. The unit-cell packing can be described as a tilted herringbone motif. The H⋯H, H⋯O/O⋯H, H⋯C/C⋯H and C⋯C contacts contribute 46.7, 24.2, 16.7 and 7.6%, respectively, to its Hirshfeld surface.

## Abstract 2

In the title coumarin derivative, the dihedral angle between the 2*H*-chromen-2-one ring system and the phenyl ring is 89.12 (5)°. In the crystal, the mol­ecules are linked by C—H⋯O hydrogen bonds into [010] double chains.

## 1. AFRAMED and chemical context

The AFRAMED (Supporting research and training in Africa through remote measurements; Abdel-Aal *et al.*, 2023 ▸) CNRS project was developed by the Chair of the IUCr Africa Initiative (Professor Claude Lecomte) and his team for Crystallography Education in Africa. The project is based on the remote control by an African laboratory of a diffractometer based in France (in fact now at CRM2) to perform X-ray single-crystal diffraction measurements for research and teaching purposes. Selected crystals are sent to the French partner by African researchers who control the data collection remotely and then receive the intensity data by e-mail. The project was launched in August 2022 and is co-financed by the French Centre National de la Recherche Scientifique (CNRS), the United Nations Educational, Scientific and Cultural Organization (UNESCO), and the Inter­national Union of Crystallography (IUCr). Two main steps define AFRAMED: first, four weeks training of African Partners (young lecturers with permanent positions) on a single-crystal diffractometer, and in the second step, the African researchers’ laboratories are focal points to assist their colleagues for remote measurements. To date, representatives of Algeria, Cameroon; Congo Brazzaville; Cote d’Ivoire, Egypt, Gabon and Senegal have been trained at the CRM2 laboratory of the Université de Lorraine, France.

This paper presents one of the results of this training: the synthesis, crystal structure and Hirshfeld surface analysis of the title coumarin derivative, **I**, synthesized by colleagues from Burkina Faso. Such coumaruin derivatives have various biological activities such as anti­cancer (Lacy *et al.*, 2004 ▸; Kostova, 2005 ▸), anti-inflammatory (Todeschini *et al.*, 1998 ▸), anti­viral (Borges *et al.*, 2005 ▸), anti-malarial (Agarwal *et al.*, 2005 ▸), anti-glaucoma (Ziki *et al.*, 2023 ▸) and anti­coagulant (Maurer *et al.*, 1998 ▸) properties. 
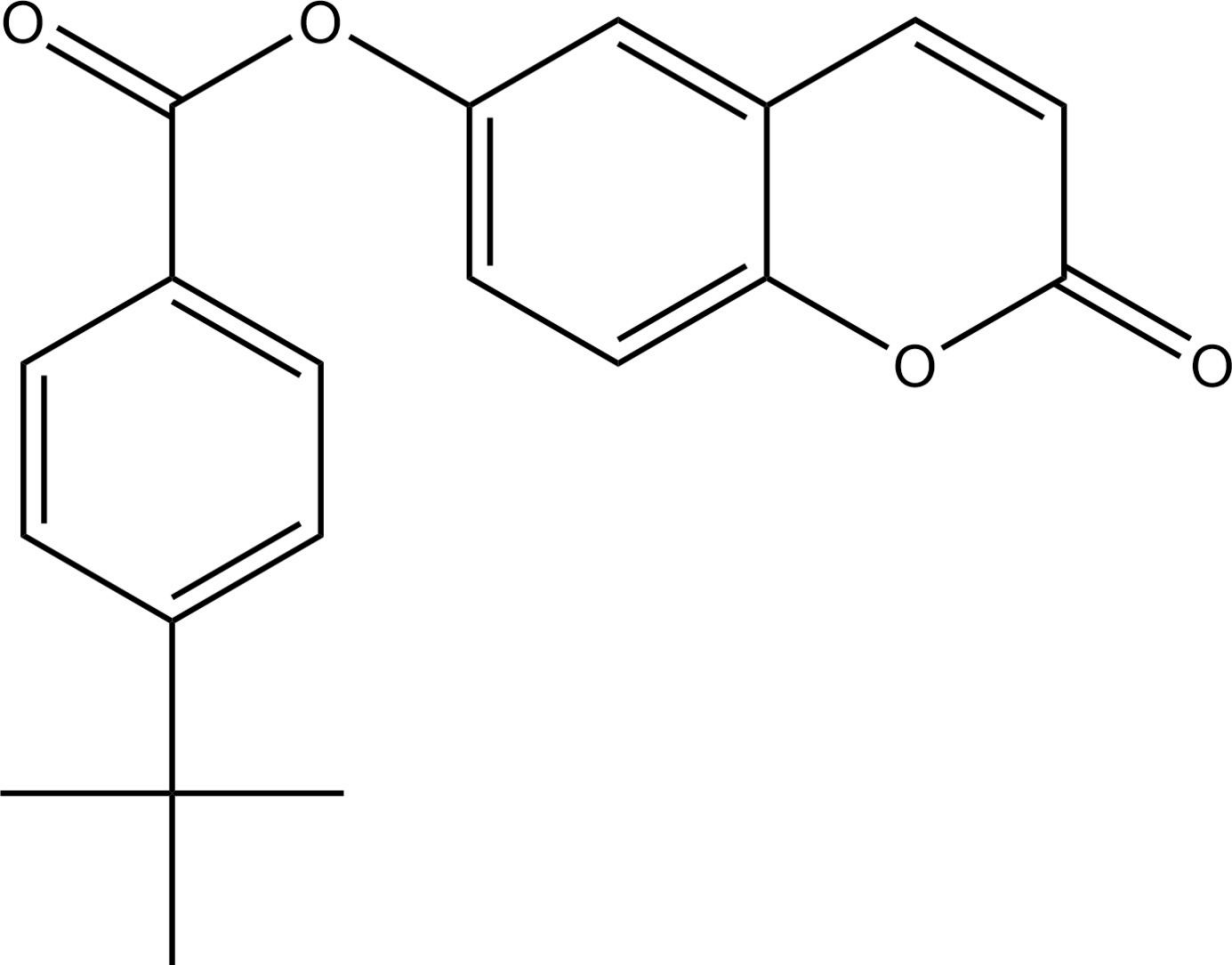


## 2. Structural commentary

**Figure 1:**
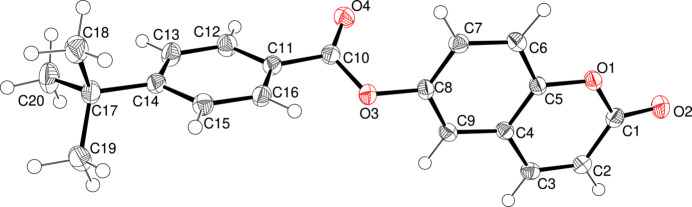
The mol­ecular structure of **I** with displacement ellipsoids drawn at the 50% probability level.

As shown in Fig. 1 ▸, the C1–C9/O1/O2 2*H*-chromen-2-one ring system of **I** is almost planar (r.m.s. deviation = 0.044 Å) and the dihedral angle between this ring system and the C11–C16 phenyl group in the 4-*tert*-butyl­benzoate moiety is 89.12 (5)°. This near perpendicular orientation has been observed in other coumarin derivatives with the same motif (Ji *et al.*, 2016 ▸). The dihedral angles between the linking C10/C11/O3/O4 ester group and the pendant C1–C9/O1/O2 and C11–C16 groupings are 64.38 (5) and 25.05 (6)°, respectively, indicating that the major twist in the mol­ecule occurs about the C8—O3 bond. An inspection of the bond lengths shows that there is a slight asymmetry of electronic distribution around the coumarin ring: the difference between the C2=C3 [1.343 (2) Å] and C1—C2 [1.449 (2) Å] separations confirms the double-bond character of the former as indicated in the chemical scheme. Atom C20 of the *tert*-butyl group lies close to the plane of its attached ring [deviation = 0.226 (2) Å] whereas C18 and C19 are displaced either side of the ring [deviations = −1.465 (1) and 0.964 (1) Å, respectively].

## 3. Supra­molecular features

**Figure 2:**
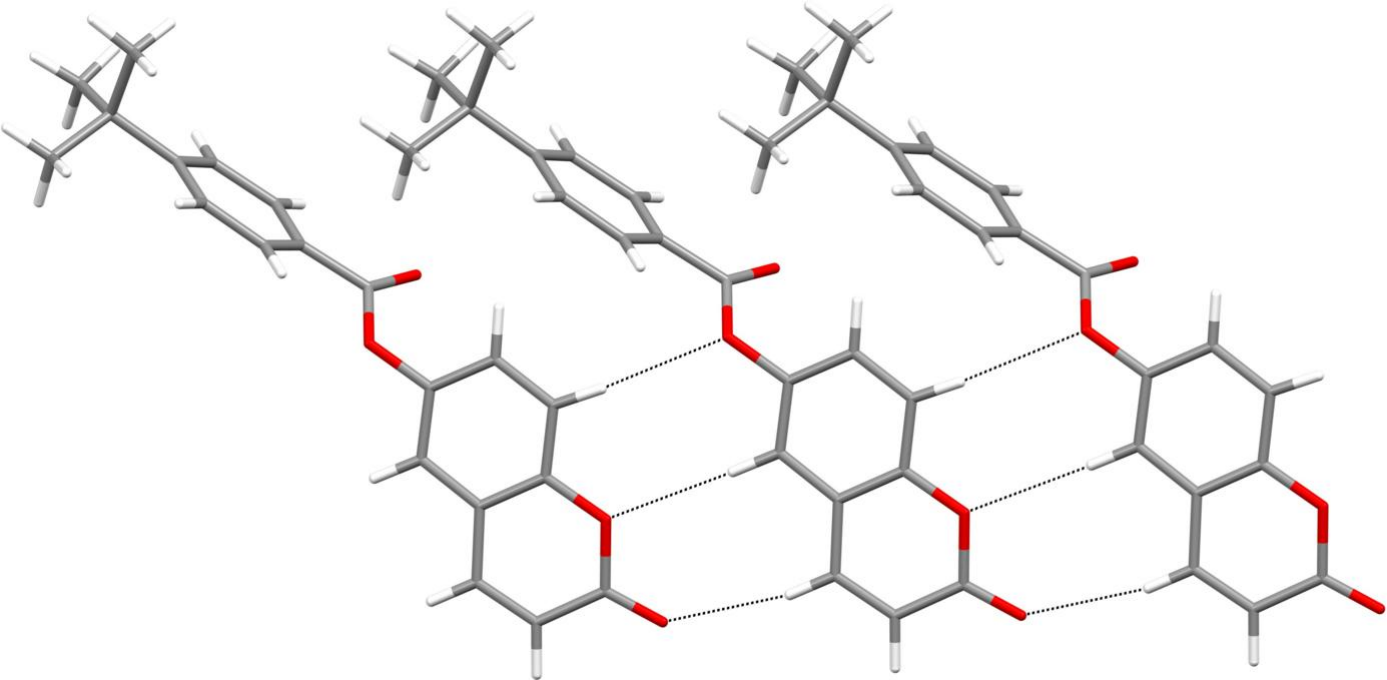
Fragment of a [010] chain in the structure of **I** showing the hydrogen bonds involving C3, C6 and C9 as black dashed lines.

**Figure 3:**
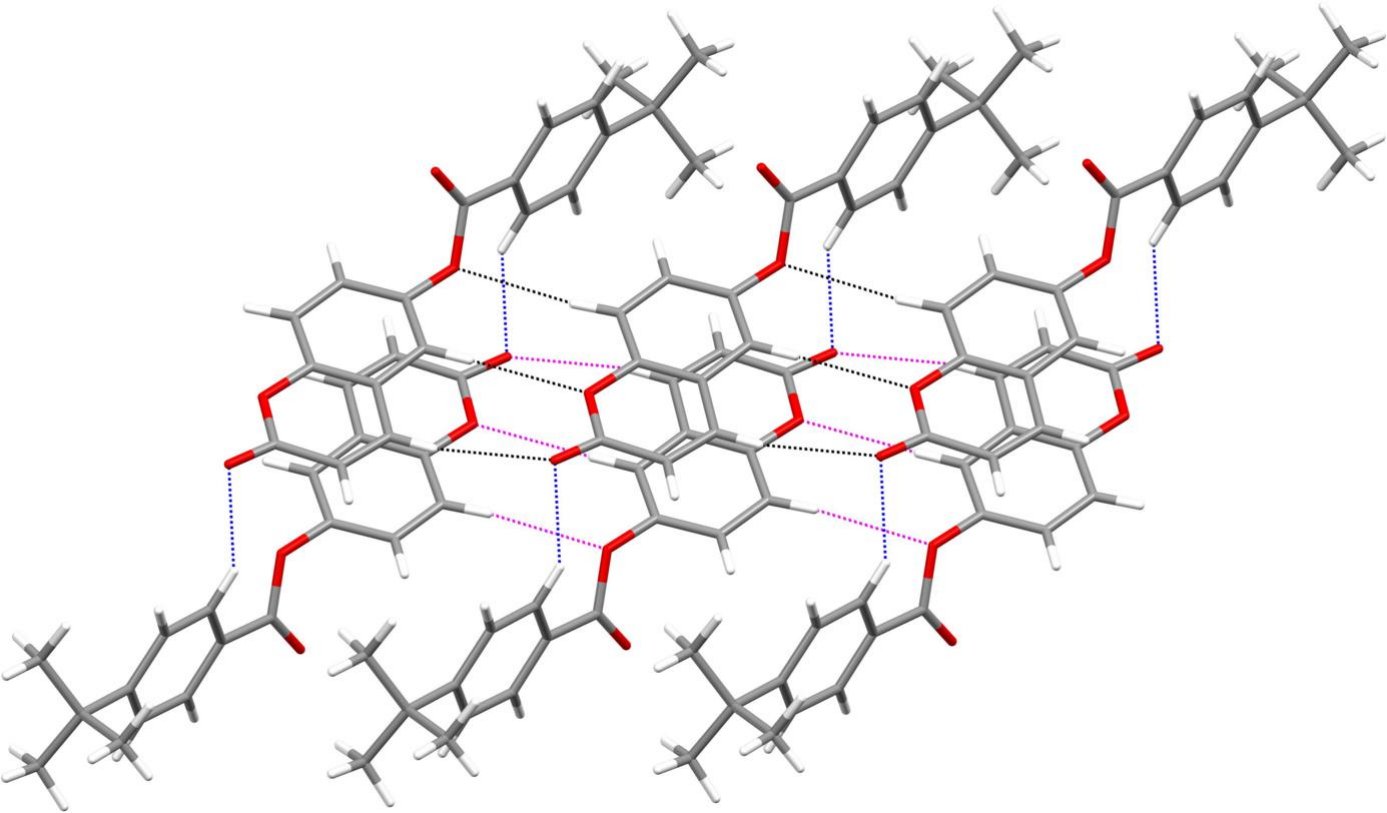
Partial packing diagram for **I** showing [010] double chains arising from C—H⋯O hydrogen bonds (black dashed lines in one chain, magenta dashed lines in the other and the C16 cross-linking bonds in blue).

**Figure 4:**
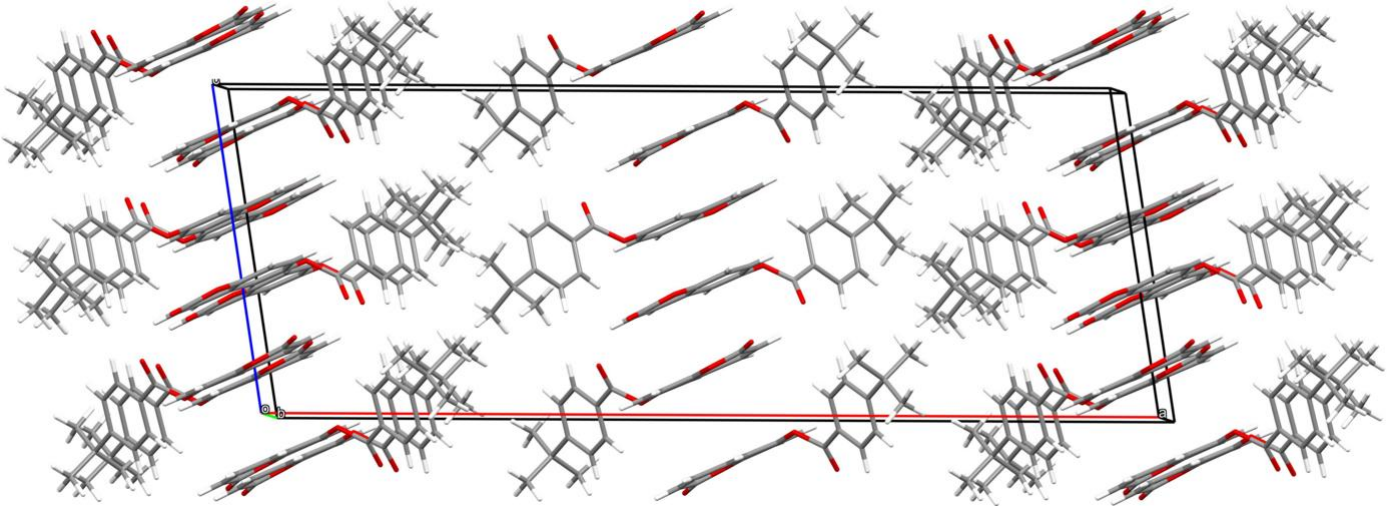
The unit-cell packing for **I** viewed down [010].

In the crystal, the mol­ecules of **I** are connected by C—H⋯O hydrogen bonds (Table 1 ▸) to build double chains propagating in the [010] direction: this motif results in two adjacent 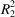 (8)loops between each pair of mol­ecules in one chain formed by the C3, C6 and C9 hydrogen bonds (Fig. 2 ▸). The C16 hydrogen bond provides the linkage to the second chain (Fig. 3 ▸). The pendant 4-*tert*-butyl­benzoate moieties are parallel and shifted by translation along the *b* axis. Aromatic π–π stacking inter­actions between centrosymmetric pairs of C4–C9 rings reinforce the cohesion of the double chains [centroid–centroid separation = 3.6301 (8), slippage = 1.579 Å]. The unit-cell packing of **I** can be described as a tilted herringbone motif (Fig. 4 ▸), as also observed in the crystal structure of 1-(1,2-di­hydro­phthalazin-1-yl­idene)-2-[1-(thio­phen-2-yl)eth­ylidene]hydrazine (Majoumo-Mbe *et al.*, 2019 ▸).

## 4. Database survey

A search of the Cambridge Structural Database (CSD, version 5.43; update 3, September 2022; Groom *et al.*, 2016 ▸) for structures having a coumarin motif similar to that of **I** returned five hits for to the following mol­ecules: 4-methyl-2-oxo-2*H*-1-benzo­pyran-6-yl pyridine-2-carboxyl­ate (CSD refcode ATOROT; Ji *et al.*, 2016 ▸), 4-methyl-2-oxo-2*H*-1-benzo­pyran-6-yl pyridine-3-carboxyl­ate (ATORUZ; Ji *et al.*, 2016 ▸), 4-methyl-2-oxo-2*H*-1-benzo­pyran-6-yl pyridine-4-carboxyl­ate (ATOSAG; Ji *et al.*, 2016 ▸), 6-acet­oxy­coumarin (GASXON; Murthy *et al.*, 1988 ▸) and 4-methyl-2-oxo-2H-chromen-6-yl benzoate (YEFSOU; Ji *et al.*, 2017 ▸). ATORUZ only features a C6—H*6*⋯O3 hydrogen bond because a methyl group is bonded to C9 (according to the numbering scheme of **I**). This prevents the formation of layers like those found in the packing of **I**, although similar layers are found in GASXON.

## 5. Hirshfeld surface and Fingerprint plots

**Figure 5:**
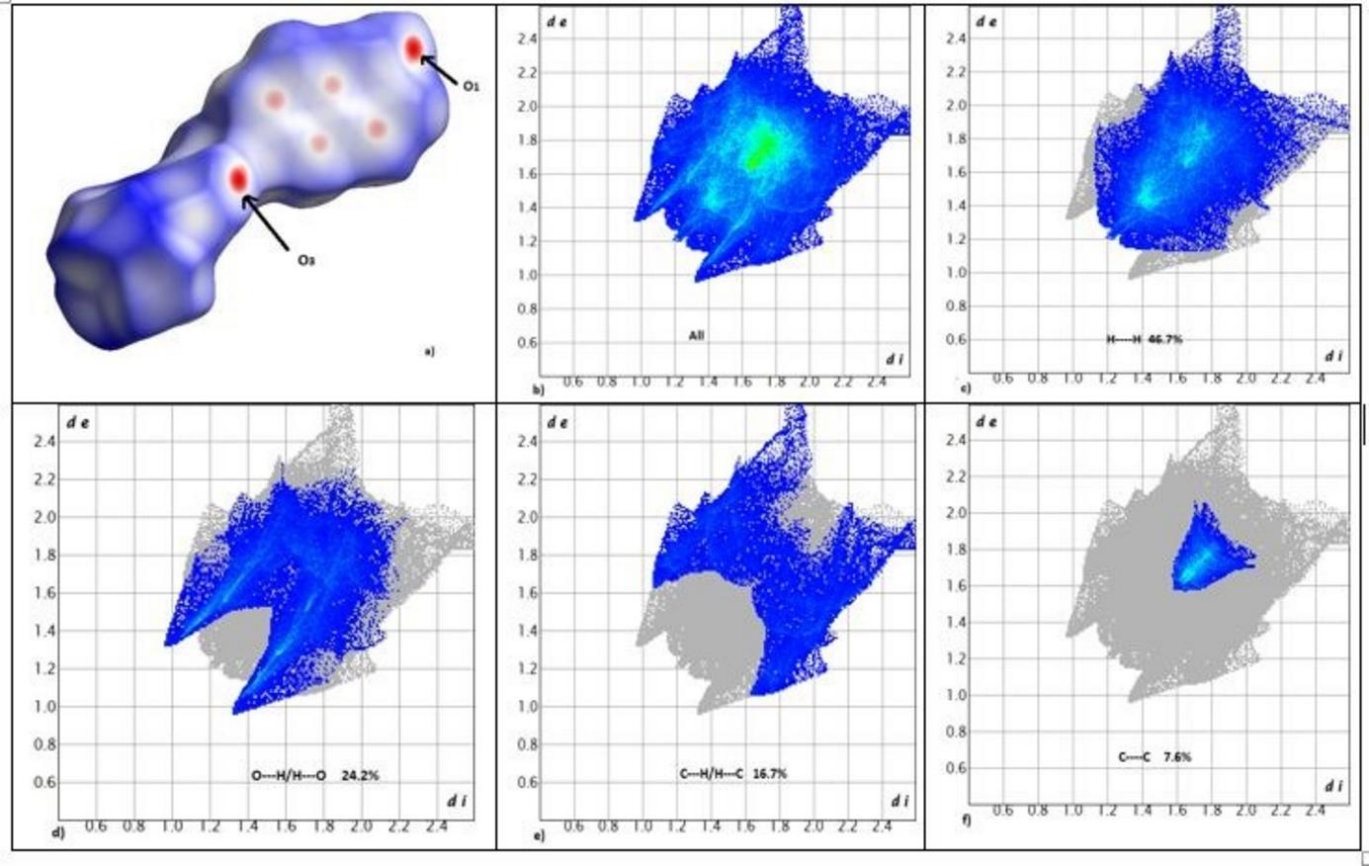
(*a*) Hirshfeld surface of **I** mapped over *d*_norm_ and (*b*) two-dimensional fingerprint plots of (*b*) overall and delineated into contributions from different contacts: (*c*) H—H, (*d*) H—O/O—H, (*e*) H—C/C—H and (*f*) C—C.

The inter­actions mentioned above are confirmed by the two-dimensional fingerprint plots of **I** (Fig. 5 ▸). The greatest contributions are the H⋯H and H⋯O/O⋯H contacts with 46.7 and 24.2%, respectively. The H⋯C/C⋯H and C⋯C contacts contribute 16.7 and 7.6%, respectively. The contributions of the H⋯H inter­actions in **I** to Hirshfeld surface are greater than those found in 2-oxo-2*H*-chromen-3-yl 4-chloro­benzoate (Ziki *et al.* 2017 ▸); this can be related to the packing of the 2*H*-1-chromen-6-yl moieties of **I**. The H⋯O/O⋯H contacts are related to the C—H⋯O1 hydrogen bonds shown in Fig. 2 ▸. Their contact points are shown in red and are labelled on the Hirshfeld surface (see Fig. 5 ▸ *a*).

## 6. Synthesis and crystallization

To 30 ml solution of 4-*tert*-butyl­benzoyl chloride (1.2 g; 6.17 mmol) in dry tetra­hydro­furan, were added dry tri­ethyl­amine (2.6 ml; 3.1 mmol) and 6-hy­droxy­coumarin (1.00 g; 6.17 mmol) in small portions over 30 min. The mixture was then refluxed for 4 h and poured into 40 ml of chloro­form. The solution was acidified with diluted hydro­chloric acid until the pH was 2.5. The organic layer was extracted, washed with water to neutrality, dried over MgSO_4_ and the solvent removed. The resulting precipitate was suction filtered, washed with petroleum ether and recrystallized from chloro­form solution to give colorless prismatic crystals of **I** in a yield of 84%.

## 7. Refinement details

Crystal data, data collection and structure refinement details are summarized in Table 2 ▸. The H atoms were located in difference maps and their positions and *U*_iso_ values were freely refined.

## Supplementary Material

Crystal structure: contains datablock(s) I. DOI: 10.1107/S2056989023011052/hb8087sup1.cif

Structure factors: contains datablock(s) I. DOI: 10.1107/S2056989023011052/hb8087Isup2.hkl

Supporting information file. DOI: 10.1107/S2056989023011052/hb8087Isup2.cml

CCDC reference: 2301781

Additional supporting information:  crystallographic information; 3D view; checkCIF report

## Figures and Tables

**Table 1:** Hydrogen-bond geometry (Å, °)

*D*—H⋯*A*	*D*—H	H⋯*A*	*D*⋯*A*	*D*—H⋯*A*
C9—H9⋯O1^i^	0.987 (13)	2.584 (13)	3.5682 (14)	174.8 (10)
C6—H6⋯O3^ii^	0.985 (14)	2.603 (15)	3.5835 (14)	173.9 (12)
C9—H9⋯O1^i^	0.987 (13)	2.584 (13)	3.5682 (14)	174.8 (10)
C16—H16⋯O2^iii^	0.968 (14)	2.416 (15)	3.2628 (16)	146.0 (11)

Symmetry codes: (i) 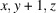 ; (ii) 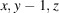 ; (iii) 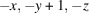 .

**Table 2:** Experimental details

Crystal data	
Chemical formula	C_20_H_18_O_4_
*M*_r_	322.34
Crystal system, space group	Monoclinic, *C*2/*c*
Temperature (K)	100
*a*, *b*, *c* (Å)	35.908 (4), 6.8473 (6), 13.2661 (11)
β (°)	98.915 (4)
*V* (Å^3^)	3222.3 (5)
*Z*	8
Radiation type	Mo *K*α
μ (mm^−1^)	0.09
Crystal size (mm)	0.20 × 0.15 × 0.08

Data collection	
Diffractometer	Bruker D8 Venture
Absorption correction	Multi-scan (*SADABS*; Krause *et al.*, 2015 ▸)
*T*_min_, *T*_max_	0.731, 0.895
No. of measured, independent and observed [*I* > 2σ(*I*)] reflections	60054, 4940, 3518
*R*_int_	0.061
(sin θ/λ)_max_ (Å^−1^)	0.716

Refinement	
*R*[*F* ^2^ > 2σ(*F* ^2^)], *wR*(*F* ^2^), *S*	0.044, 0.124, 1.11
No. of reflections	4940
No. of parameters	289
H-atom treatment	All H-atom parameters refined
Δρ_max_, Δρ_min_ (e Å^−3^)	0.30, −0.27

Computer programs: *APEX3* and *SAINT* (Bruker, 2019 ▸), *SHELXT* (Sheldrick, 2015*a* ▸), *SHELXL2018/3* (Sheldrick, 2015*b* ▸), *ORTEP-3 for Windows* (Farrugia, 2012 ▸) and *publCIF* (Westrip, 2010 ▸).

## supplementary crystallographic information

### Crystal data

**Table:**

C_20_H_18_O_4_	*F*(000) = 1360
*M**_r_* = 322.34	*D*_x_ = 1.329 Mg m^−^^3^
Monoclinic, *C*2/*c*	Mo *K*α radiation, λ = 0.71073 Å
Hall symbol: -C 2yc	Cell parameters from 4940 reflections
*a* = 35.908 (4) Å	θ = 2.3–30.6°
*b* = 6.8473 (6) Å	µ = 0.09 mm^−^^1^
*c* = 13.2661 (11) Å	*T* = 100 K
β = 98.915 (4)°	Prism, colourless
*V* = 3222.3 (5) Å^3^	0.20 × 0.15 × 0.08 mm
*Z* = 8	

### Data collection

**Table:**

Bruker D8 Venture diffractometer	4940 independent reflections
Radiation source: fine-focus sealed tube	3518 reflections with *I* > 2σ(*I*)
Mirror monochromator	*R*_int_ = 0.061
φ and ω scans	θ_max_ = 30.6°, θ_min_ = 2.3°
Absorption correction: multi-scan (*SADABS*; Krause *et al.*, 2015)	*h* = −51→51
*T*_min_ = 0.731, *T*_max_ = 0.895	*k* = −9→9
60054 measured reflections	*l* = −18→18

### Refinement

**Table:**

Refinement on *F*^2^	Primary atom site location: structure-invariant direct methods
Least-squares matrix: full	Secondary atom site location: structure-invariant direct methods
*R*[*F*^2^ > 2σ(*F*^2^)] = 0.044	Hydrogen site location: difference Fourier map
*wR*(*F*^2^) = 0.124	All H-atom parameters refined
*S* = 1.11	*w* = 1/[σ^2^(*F*_o_^2^) + (0.0582*P*)^2^ + 0.7544*P*] where *P* = (*F*_o_^2^ + 2*F*_c_^2^)/3
4940 reflections	(Δ/σ)_max_ < 0.001
289 parameters	Δρ_max_ = 0.30 e Å^−^^3^
0 restraints	Δρ_min_ = −0.27 e Å^−^^3^

### Special details

**Table:**

Geometry. All esds (except the esd in the dihedral angle between two l.s. planes) are estimated using the full covariance matrix. The cell esds are taken into account individually in the estimation of esds in distances, angles and torsion angles; correlations between esds in cell parameters are only used when they are defined by crystal symmetry. An approximate (isotropic) treatment of cell esds is used for estimating esds involving l.s. planes.

### Fractional atomic coordinates and isotropic or equivalent isotropic displacement parameters (Å^2^)

**Table:**

	*x*	*y*	*z*	*U*_iso_*/*U*_eq_	
O1	0.01365 (2)	0.14244 (11)	0.13861 (6)	0.02265 (19)	
O3	−0.07925 (2)	0.77690 (11)	0.03962 (6)	0.02359 (19)	
C9	−0.01929 (3)	0.65096 (16)	0.10952 (8)	0.0204 (2)	
O2	0.06636 (3)	0.00843 (12)	0.21536 (7)	0.0293 (2)	
O4	−0.11921 (2)	0.70415 (12)	0.15117 (7)	0.0276 (2)	
C5	−0.00869 (3)	0.30453 (15)	0.11373 (8)	0.0195 (2)	
C2	0.06565 (3)	0.35501 (17)	0.20298 (9)	0.0225 (2)	
C6	−0.04569 (3)	0.26950 (17)	0.07004 (9)	0.0219 (2)	
C4	0.00547 (3)	0.49326 (15)	0.13275 (8)	0.0192 (2)	
C1	0.05017 (3)	0.15982 (17)	0.18837 (9)	0.0227 (2)	
C10	−0.11073 (3)	0.80567 (16)	0.08412 (9)	0.0222 (2)	
C3	0.04453 (3)	0.51431 (17)	0.17689 (9)	0.0211 (2)	
C11	−0.13230 (3)	0.97493 (16)	0.03591 (9)	0.0221 (2)	
C7	−0.06982 (3)	0.42622 (17)	0.04737 (9)	0.0221 (2)	
C8	−0.05615 (3)	0.61479 (16)	0.06872 (9)	0.0207 (2)	
C16	−0.12922 (3)	1.03226 (17)	−0.06341 (9)	0.0231 (2)	
C14	−0.17523 (3)	1.28898 (17)	−0.05714 (9)	0.0235 (2)	
C15	−0.15077 (3)	1.18512 (17)	−0.10943 (9)	0.0236 (2)	
C12	−0.15665 (4)	1.07610 (18)	0.08908 (10)	0.0272 (3)	
C13	−0.17742 (4)	1.23237 (18)	0.04322 (10)	0.0280 (3)	
C17	−0.19912 (3)	1.45327 (17)	−0.11186 (10)	0.0263 (3)	
C19	−0.17461 (4)	1.58787 (19)	−0.16687 (12)	0.0327 (3)	
C20	−0.21829 (5)	1.5761 (2)	−0.03849 (13)	0.0402 (4)	
C18	−0.22939 (4)	1.3594 (2)	−0.19155 (13)	0.0375 (3)	
H9	−0.0100 (4)	0.7858 (19)	0.1225 (9)	0.018 (3)*	
H3	0.0540 (4)	0.644 (2)	0.1881 (10)	0.022 (3)*	
H2	0.0923 (4)	0.3644 (18)	0.2369 (9)	0.019 (3)*	
H6	−0.0534 (4)	0.132 (2)	0.0585 (11)	0.031 (4)*	
H16	−0.1128 (4)	0.963 (2)	−0.1025 (11)	0.028 (4)*	
H7	−0.0961 (4)	0.4084 (19)	0.0178 (10)	0.024 (3)*	
H15	−0.1483 (4)	1.220 (2)	−0.1788 (11)	0.028 (4)*	
H12	−0.1584 (4)	1.038 (2)	0.1596 (12)	0.033 (4)*	
H13	−0.1935 (4)	1.302 (2)	0.0848 (12)	0.040 (4)*	
H19A	−0.1646 (5)	1.519 (2)	−0.2236 (13)	0.042 (4)*	
H18A	−0.2457 (5)	1.460 (2)	−0.2292 (12)	0.042 (4)*	
H19B	−0.1528 (5)	1.642 (2)	−0.1159 (13)	0.046 (5)*	
H20A	−0.2378 (4)	1.495 (2)	−0.0068 (12)	0.038 (4)*	
H18B	−0.2453 (5)	1.273 (3)	−0.1618 (14)	0.054 (5)*	
H20B	−0.1987 (4)	1.634 (2)	0.0198 (12)	0.041 (4)*	
H20C	−0.2317 (5)	1.687 (3)	−0.0776 (13)	0.051 (5)*	
H19C	−0.1898 (5)	1.702 (2)	−0.1991 (12)	0.045 (4)*	
H18C	−0.2170 (5)	1.281 (3)	−0.2455 (14)	0.055 (5)*	

### Atomic displacement parameters (Å^2^)

**Table:**

	*U*^11^	*U*^22^	*U*^33^	*U*^12^	*U*^13^	*U*^23^
O1	0.0223 (4)	0.0180 (4)	0.0272 (4)	0.0017 (3)	0.0023 (3)	0.0003 (3)
O3	0.0218 (4)	0.0213 (4)	0.0278 (4)	0.0041 (3)	0.0043 (3)	0.0037 (3)
C9	0.0228 (6)	0.0186 (5)	0.0200 (5)	−0.0004 (4)	0.0039 (4)	0.0008 (4)
O2	0.0292 (5)	0.0237 (4)	0.0349 (5)	0.0074 (3)	0.0046 (4)	0.0034 (4)
O4	0.0293 (5)	0.0255 (4)	0.0289 (5)	0.0011 (4)	0.0069 (4)	0.0031 (3)
C5	0.0218 (5)	0.0173 (5)	0.0197 (5)	0.0018 (4)	0.0044 (4)	0.0010 (4)
C2	0.0200 (5)	0.0251 (6)	0.0227 (6)	0.0001 (4)	0.0042 (4)	−0.0004 (4)
C6	0.0250 (6)	0.0184 (5)	0.0223 (6)	−0.0014 (4)	0.0035 (4)	−0.0009 (4)
C4	0.0215 (5)	0.0182 (5)	0.0178 (5)	−0.0005 (4)	0.0033 (4)	0.0007 (4)
C1	0.0219 (5)	0.0245 (6)	0.0225 (6)	0.0027 (4)	0.0058 (4)	0.0003 (4)
C10	0.0210 (5)	0.0211 (5)	0.0239 (6)	−0.0005 (4)	0.0017 (4)	−0.0027 (4)
C3	0.0223 (6)	0.0215 (5)	0.0199 (5)	−0.0023 (4)	0.0045 (4)	−0.0003 (4)
C11	0.0197 (5)	0.0206 (5)	0.0256 (6)	0.0001 (4)	0.0023 (4)	−0.0012 (4)
C7	0.0206 (6)	0.0226 (5)	0.0226 (6)	−0.0012 (4)	0.0021 (4)	0.0005 (4)
C8	0.0226 (6)	0.0188 (5)	0.0209 (5)	0.0038 (4)	0.0042 (4)	0.0017 (4)
C16	0.0211 (6)	0.0227 (5)	0.0258 (6)	0.0020 (4)	0.0043 (4)	−0.0019 (4)
C14	0.0190 (5)	0.0207 (5)	0.0297 (6)	−0.0001 (4)	0.0010 (4)	−0.0014 (4)
C15	0.0233 (6)	0.0240 (6)	0.0228 (6)	0.0007 (4)	0.0015 (4)	−0.0001 (4)
C12	0.0288 (6)	0.0282 (6)	0.0257 (6)	0.0037 (5)	0.0072 (5)	0.0014 (5)
C13	0.0275 (6)	0.0277 (6)	0.0299 (6)	0.0067 (5)	0.0081 (5)	−0.0014 (5)
C17	0.0232 (6)	0.0229 (6)	0.0318 (7)	0.0041 (5)	0.0008 (5)	−0.0002 (5)
C19	0.0331 (7)	0.0242 (6)	0.0396 (8)	0.0013 (5)	0.0017 (6)	0.0042 (5)
C20	0.0427 (9)	0.0341 (7)	0.0451 (9)	0.0169 (7)	0.0104 (7)	0.0018 (6)
C18	0.0288 (7)	0.0307 (7)	0.0482 (9)	0.0014 (6)	−0.0094 (6)	0.0017 (6)

### Geometric parameters (Å, º)

**Table:**

O1—C1	1.3789 (14)	C7—H7	0.973 (13)
O1—C5	1.3791 (13)	C16—C15	1.3858 (16)
O3—C10	1.3680 (14)	C16—H16	0.967 (14)
O3—C8	1.4036 (13)	C14—C15	1.3956 (17)
C9—C8	1.3724 (16)	C14—C13	1.4005 (18)
C9—C4	1.4023 (15)	C14—C17	1.5281 (16)
C9—H9	0.987 (13)	C15—H15	0.967 (14)
O2—C1	1.2150 (14)	C12—C13	1.3895 (17)
O4—C10	1.2047 (14)	C12—H12	0.983 (15)
C5—C6	1.3855 (16)	C13—H13	0.983 (16)
C5—C4	1.3972 (15)	C17—C20	1.5286 (19)
C2—C3	1.3430 (16)	C17—C19	1.5347 (19)
C2—C1	1.4490 (16)	C17—C18	1.5348 (18)
C2—H2	0.993 (13)	C19—H19A	0.999 (17)
C6—C7	1.3829 (16)	C19—H19B	1.021 (17)
C6—H6	0.985 (14)	C19—H19C	1.007 (17)
C4—C3	1.4410 (16)	C20—H20A	1.034 (16)
C10—C11	1.4829 (16)	C20—H20B	1.040 (16)
C3—H3	0.956 (13)	C20—H20C	0.999 (18)
C11—C12	1.3903 (17)	C18—H18A	0.990 (16)
C11—C16	1.3955 (17)	C18—H18B	0.948 (18)
C7—C8	1.3949 (16)	C18—H18C	1.047 (18)
			
C1—O1—C5	121.32 (9)	C11—C16—H16	120.8 (8)
C10—O3—C8	119.20 (9)	C15—C14—C13	117.63 (11)
C8—C9—C4	119.17 (10)	C15—C14—C17	119.29 (11)
C8—C9—H9	121.0 (7)	C13—C14—C17	123.04 (11)
C4—C9—H9	119.8 (7)	C16—C15—C14	121.16 (11)
O1—C5—C6	116.43 (9)	C16—C15—H15	118.5 (8)
O1—C5—C4	121.28 (10)	C14—C15—H15	120.3 (8)
C6—C5—C4	122.28 (10)	C13—C12—C11	119.91 (12)
C3—C2—C1	121.66 (11)	C13—C12—H12	120.9 (8)
C3—C2—H2	122.0 (7)	C11—C12—H12	119.2 (8)
C1—C2—H2	116.3 (7)	C12—C13—C14	121.60 (11)
C7—C6—C5	118.98 (10)	C12—C13—H13	116.8 (9)
C7—C6—H6	123.8 (8)	C14—C13—H13	121.6 (9)
C5—C6—H6	117.2 (8)	C14—C17—C20	112.19 (11)
C5—C4—C9	118.16 (10)	C14—C17—C19	110.30 (10)
C5—C4—C3	118.03 (10)	C20—C17—C19	108.67 (11)
C9—C4—C3	123.80 (10)	C14—C17—C18	107.72 (10)
O2—C1—O1	116.33 (10)	C20—C17—C18	109.17 (12)
O2—C1—C2	126.25 (11)	C19—C17—C18	108.73 (12)
O1—C1—C2	117.41 (10)	C17—C19—H19A	111.9 (9)
O4—C10—O3	123.78 (10)	C17—C19—H19B	109.9 (9)
O4—C10—C11	126.47 (11)	H19A—C19—H19B	110.0 (13)
O3—C10—C11	109.74 (10)	C17—C19—H19C	110.8 (9)
C2—C3—C4	119.92 (10)	H19A—C19—H19C	106.1 (13)
C2—C3—H3	122.8 (8)	H19B—C19—H19C	107.9 (13)
C4—C3—H3	117.2 (8)	C17—C20—H20A	111.2 (9)
C12—C11—C16	119.14 (11)	C17—C20—H20B	111.4 (9)
C12—C11—C10	119.88 (11)	H20A—C20—H20B	109.1 (13)
C16—C11—C10	120.97 (10)	C17—C20—H20C	108.3 (10)
C6—C7—C8	119.02 (11)	H20A—C20—H20C	108.6 (14)
C6—C7—H7	121.8 (8)	H20B—C20—H20C	108.1 (13)
C8—C7—H7	119.2 (8)	C17—C18—H18A	110.9 (9)
C9—C8—C7	122.33 (10)	C17—C18—H18B	112.4 (11)
C9—C8—O3	117.29 (10)	H18A—C18—H18B	107.1 (14)
C7—C8—O3	120.12 (10)	C17—C18—H18C	110.8 (10)
C15—C16—C11	120.53 (11)	H18A—C18—H18C	107.2 (13)
C15—C16—H16	118.6 (8)	H18B—C18—H18C	108.3 (14)
			
C1—O1—C5—C6	−175.80 (10)	C5—C6—C7—C8	0.31 (17)
C1—O1—C5—C4	3.67 (16)	C4—C9—C8—C7	−1.38 (17)
O1—C5—C6—C7	177.35 (10)	C4—C9—C8—O3	−175.41 (10)
C4—C5—C6—C7	−2.11 (17)	C6—C7—C8—C9	1.43 (18)
O1—C5—C4—C9	−177.29 (10)	C6—C7—C8—O3	175.30 (10)
C6—C5—C4—C9	2.15 (17)	C10—O3—C8—C9	−120.25 (11)
O1—C5—C4—C3	1.60 (16)	C10—O3—C8—C7	65.57 (14)
C6—C5—C4—C3	−178.97 (10)	C12—C11—C16—C15	−1.48 (18)
C8—C9—C4—C5	−0.39 (16)	C10—C11—C16—C15	177.09 (10)
C8—C9—C4—C3	−179.21 (10)	C11—C16—C15—C14	1.79 (18)
C5—O1—C1—O2	173.38 (10)	C13—C14—C15—C16	−0.42 (17)
C5—O1—C1—C2	−6.99 (15)	C17—C14—C15—C16	−178.56 (11)
C3—C2—C1—O2	−175.08 (12)	C16—C11—C12—C13	−0.18 (18)
C3—C2—C1—O1	5.34 (17)	C10—C11—C12—C13	−178.76 (11)
C8—O3—C10—O4	3.51 (16)	C11—C12—C13—C14	1.6 (2)
C8—O3—C10—C11	−175.37 (9)	C15—C14—C13—C12	−1.26 (18)
C1—C2—C3—C4	−0.26 (17)	C17—C14—C13—C12	176.81 (12)
C5—C4—C3—C2	−3.21 (16)	C15—C14—C17—C20	−168.91 (12)
C9—C4—C3—C2	175.60 (11)	C13—C14—C17—C20	13.06 (17)
O4—C10—C11—C12	24.57 (18)	C15—C14—C17—C19	−47.60 (15)
O3—C10—C11—C12	−156.59 (11)	C13—C14—C17—C19	134.37 (13)
O4—C10—C11—C16	−153.99 (12)	C15—C14—C17—C18	70.93 (15)
O3—C10—C11—C16	24.85 (15)	C13—C14—C17—C18	−107.11 (14)

### Hydrogen-bond geometry (Å, º)

**Table:**

*D*—H···*A*	*D*—H	H···*A*	*D*···*A*	*D*—H···*A*
C9—H9···O1^i^	0.987 (13)	2.584 (13)	3.5682 (14)	174.8 (10)
C6—H6···O3^ii^	0.985 (14)	2.603 (15)	3.5835 (14)	173.9 (12)
C9—H9···O1^i^	0.987 (13)	2.584 (13)	3.5682 (14)	174.8 (10)
C16—H16···O2^iii^	0.968 (14)	2.416 (15)	3.2628 (16)	146.0 (11)

Symmetry codes: (i) *x*, *y*+1, *z*; (ii) *x*, *y*−1, *z*; (iii) −*x*, −*y*+1, −*z*.
